# Bridging the Gap Between RCTs and RWE Through Endpoint Selection

**DOI:** 10.1007/s43441-020-00193-5

**Published:** 2020-07-06

**Authors:** Robert J. LoCasale, Chris L. Pashos, Ben Gutierrez, Nancy A. Dreyer, Toby Collins, Alan Calleja, Michael J. Seewald, Jonathan M. Plumb, Johan Liwing, Maurille Feudjo Tepie, Sajan Khosla

**Affiliations:** 1 Real World Evidence, Sanofi, Bridgewater, NJ USA; 2 AbbVie, Chicago, IL USA; 3 GSK, Philadelphia, PA USA; 4 IQVIA, Real-World Solutions, Cambridge, MA USA; 5 IQVIA, Real-World Solutions, London, UK; 6 Novartis Pharma AG/Pharma Medical Affairs, Basel, Switzerland; 7 Ferring Pharmaceuticals SA, St Prex, Switzerland; 8 CellProtect Nordic Pharmaceuticals, Stockholm, Sweden; 9 Center for Observational Research, Amgen, Cambridge, UK; 10 Real-World Evidence Center of Excellence, AstraZeneca, Cambridge, UK

## Abstract

This commentary is authored by several industry real-world evidence (RWE) experts, with support from IQVIA, as part of the 'RWE Leadership Forum': a group of Industry Leaders who have come together as non-competitive partners to understand and respond to RWD/E challenges and opportunities with a single expert voice. Here, the forum discusses the value in bridging the industry disconnect between RTCs and RWE, with a view to promoting the use of RWE in the RCT environment. RCT endpoints are explored along several axes including their clinical relevance and their measure of direct patient benefit, and then compared with their real-world counterparts to identify suitable paths, or gaps, for assimilating RWE endpoints into the RCT environment.

## Introduction

What underlies the choice of a study endpoint? In today’s healthcare industry, this decision is highly nuanced and influenced by a host of factors including the study’s intended audience (regulatory, health technology assessment (HTA)/payer, HCP or patient) or the provenance of data utilized. Mounting evidence pressures on drug manufacturers to differentiate their products in congested therapeutic markets can necessitate demonstrating more than drug safety and efficacy data alone. Here, real-world evidence (RWE) with appropriately selected endpoints has the potential to support key regulatory and market access decisions by broadening the value message of a medicine with evidence of real-world effectiveness, including direct assessment of patient benefit-risk. As the real-world data (RWD) landscape advances, RCTs that are complemented or supplemented with RWE have potential to generate evidence for an expanded audience of stakeholders, from regulatory bodies to patients [1]. Moreover, RWD that is “fit for purpose” (i.e. sufficiently comprehensive and complete to answer research questions and objectives) and readily accessible has potential to reduce the data collection and cost burdens of traditional RCTs that are time-consuming and expensive to conduct. Yet, as it stands, there is still considerable disconnect between the acceptance of RCTs and RWE for regulatory and market access applications, with RWE commonly viewed as less trustworthy, perhaps as a result of employing different patient populations or study endpoints that can offer differing evidentiary perspectives [2].

Traditionally, randomized clinical trials (RCTs) provide evidence of an investigational drug’s efficacy and initial safety, but highly controlled conditions can come at the expense of generalisability into the real-world, and occasionally, relatability to routine practice. Direct or clinical efficacy endpoints in RCTs, such as overall survival or incidence of cardiovascular events, capture if people feel or function better or live longer during an assessment [3], and typically reflect clinically relevant touchpoints of a disease’s natural history. However, given the current cost and time pressures of clinical drug development, direct endpoints are not always a viable option for study. Here, the adoption of surrogate endpoints (signals that occur earlier in a disease course or more reliably across a patient populations) particularly by regulatory bodies, has brought about faster and more cost-effective clinical trials that have expedited patient access to medicines in the face of rapidly evolving therapeutic landscapes.

At the same time, some surrogate endpoints do not consistently resonate with all healthcare audiences, including healthcare providers (HCPs) and patients, as they can be perceived as a shift away from directly assessing how a patient feels or functions. Notably, this shift can also influence HTA/payor decision-makers, who in today’s climate are increasingly motivated by evidence of true clinical benefit-risk to patients and not a benefit-risk that has been proxied. These attitudes are reflected by some bodies expressly codifying a preference for evidence that informs on the “therapeutic effect relevant to the patient, in particular with regard to improving the state of health, shortening the duration of the illness, prolonging survival, reducing side effects or improving the quality of life” [4].

Similarly, RCT surrogate endpoints can stray away from being relatable to routine clinical practice, as is evident for measures derived for application in trials that require specialized data collection or implement complex scales. Under such circumstances, the context of the trial may be less relevant to ordinary practitioners and patients; a situation that potentially disadvantages all industry stakeholders, with manufacturers missing out on more targeted RCTs and regulator and HTAs/payers having to put evidence into perspective.

It is at this juncture that the healthcare industry is now recognising the potential of RWD as a source of clinically meaningful evidence for consideration by key industry decision-makers, including when embedded within traditional RCT assessments [5, 6]. The value of RWE is attributed to the fact that data collection is undertaken for routine care and practice, making RWD creation essentially free of charge with the added benefit of being representative of typical patients, events and outcomes. In contrast to 5–7 years ago, RWE is now being perceived as more than just a solution for post-approval safety commitments, with a wealth of submissions now bringing RWE complemented RCTs (with RWE endpoints) to the frontline of drug approvals and market access [7].

Among cases put before regulatory bodies, a growing methodology for integrating RWE into the RCT setting is the use of RWD-built external comparators for single-arm trial designs. In 2017, Biomarin conducted a single-arm trial of Brineura in late infantile neuronal ceroid lipofuscinosis type 2 (Batten disease), that derived real-world motor and language endpoints (Hamburg Motor and Language Scale) for an external comparator cohort of 42 untreated patients, who served as a control group to the active arm of the trial [8, 9]. In a larger example from 2017, Amgen used retrospective analysis of historical data to generate overall survival estimations of 1139 acute lymphoblastic leukemia patients, from the US and EU. This external comparator cohort was matched on prior treatment exposure to 185 trial patients in receipt of blinatumomab for an open-label single-arm trial [10, 11]. In anticipation of further industry uptake of RWE, particularly in the regulatory setting, industry stakeholders have developed a series of frameworks and methodologies to ensure that future RWD generation is right for these applications [12, 13], with draft non-binding recommendations for submitting RWD/RWE documents made available by the FDA in 2019 [14].

Well-sourced RWD, that is fit-for-purpose and handled with appropriate methodologies can offer transformational opportunities for RCTs, but the decisions on how to integrate RWE into the clinical trial environment, and with which real-world endpoints, remain the subject of discussion. Here, a useful starting point is to appreciate endpoints from both RCTs and RWE over the domains that they are found to align or misalign (e.g. real-world availability, measure of clinical benefit, relevance to routine clinical practice etc.), in turn providing the insight into how and why valuable RWE endpoints can be worked into evidence generation destined for key stages of the pharma pipeline. Notably, there are areas where both RCT and real-world endpoints may fall short of giving the relevant insight to stakeholders, and here we discuss how both can move towards harmonisation, particularly in the interest of patient centricity.

## When RCT and RWE Endpoints Align

**Figure 1.**
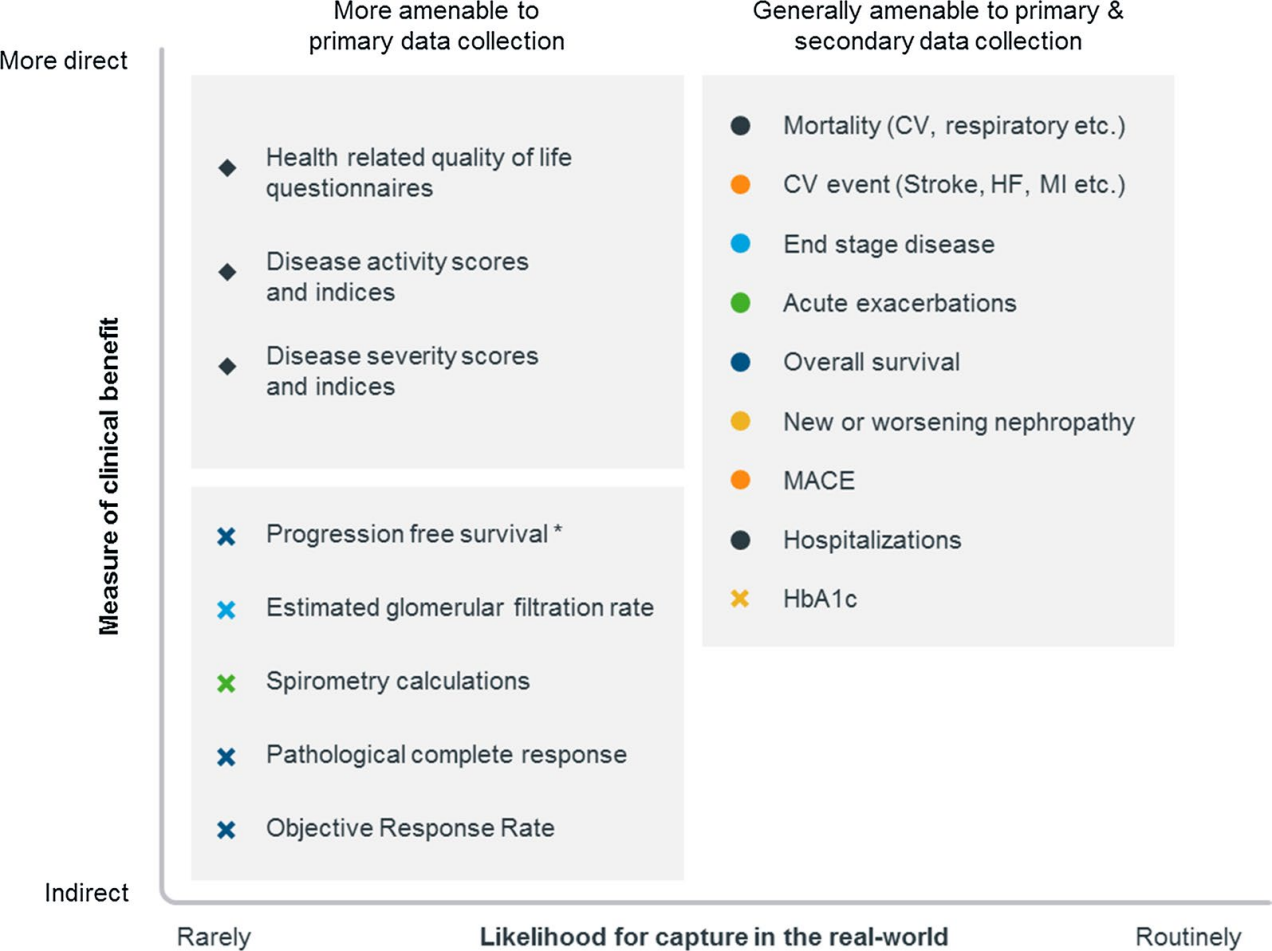
The Assessment of Clinical Trial Endpoints Along the Axes of Real-World Availability and Measure of Clinical Benefit. Common endpoints examined from cardiovascular disease (orange), chronic kidney disease (light blue), diabetes (yellow), oncology (dark blue), respiratory (green), and general disease assessments (black). Types of endpoint illustrated include: direct or clinical (circles), surrogate (crosses), and patient-centric (diamonds). *CV* Cardiovascular, *MACE* major adverse cardiovascular event, *HbA1c* haemoglobin A1c, *HF* heart failure, *MI* myocardial infarction. *Progression free survival when measured by RECIST

Many therapeutic areas already possess a degree of commonality between their real-world and RCT endpoints in terms of their relevance to stakeholders. Incidence of cardiovascular events such as myocardial infarction, heart failure and mortality are examples of endpoints adopted by RCTs and RWE that directly reflect both patient outcomes and routine clinical practice. As previously mentioned, the capacity of RWE to deliver valuable clinically relevant endpoints relates to RWD capture being tied to relevant healthcare settings [15]. However, as we look to relate more real-world endpoints into the RCT environment, it is also important to get a sense of the availability of suitable RWD signals, or ‘correlates’, generated by routine clinical practice that can serve as real-world endpoints in the RCT setting (Fig. 1). Indeed, much of the value we are now witnessing with RWE is through studies that have successfully implemented accessible RWE endpoints in an RCT setting, and that are highly relevant to routine clinical practices.

With this good degree of alignment, there are several options to integrate RWE with RCTs. In addition to external comparator cohorts, there is also opportunity to collect corresponding EMR data from trial participants or implement long-term follow-up studies that begin at the RCT level and continue into expanded real-world populations. With access to EMR, RWD supported RCTs could gain access to a broader set of information for assessing the risk-benefit profile of drugs and potentially reduce the burden of data collection from RCTs. As an added benefit, this could increase the external validity of RCTs as more real-world features are incorporated into the study design, possibly providing access to research interests that RCTs typically do not set out to answer. These include expanded efficacy, tolerability of therapies in non-target populations and comparative effectiveness against existing products or the current standard of care. Furthermore, where it is impractical or unethical to implement a placebo control arm in an RCT, or to have a control arm at all, RWD external comparator cohorts deliver valuable control populations based on current standards of care.

The other opportunity that arises with readily available RWE is the chance to understand and contextualize the relationship between outcomes from clinical trials and RWE that utilize comparable endpoints [16–18]. Although this is beyond the scope of this discussion, it is important to recognize that real-world studies that fail to reproduce outcomes of analogous RCT study designs are not automatically incorrect, but could very well represent evidence from another standpoint, as is often the case with real-world effectiveness.

## Opportunities Associated with the Misalignment of RCT and RWE Endpoints

Equally as noteworthy are the potential evidence opportunities precipitated by RCT/RWE endpoint misalignment, a paradigm arising when RCT endpoints have low real-world availability (in some instances due to low relevance to routine clinical practice), or differences in their capacity to signal direct clinical benefits. Here, surrogate endpoints provide one arena for demonstrating how hybrid methodologies could enhance RCTs that employ endpoints with narrower stakeholder appeal. For example, RECIST-based oncology endpoints such as progression free survival (PFS) and objective response rate (OOR), are not followed in the real-world in the same manner as they are collected in clinical trials, so they are generally unavailable in RWD, albeit in the rarer instances where they can be derived [19].

Another example is the use of clinically relevant surrogate spirometry endpoints in trials for respiratory disease such as chronic obstructive pulmonary disorder (COPD) or idiopathic pulmonary fibrosis (IPF) [20], that contrast with RWE endpoints of all-cause mortality or hospitalisations. In trials, the preference of the surrogate endpoint partially relates to the convenience of spirometry, that comes at the expense of direct relevance to clinical outcomes. Conversely, the risk of relying solely on hospitalisations or mortality endpoints is the uncertainty that patients will experience the endpoint over the duration of the trial, potentially reducing the statistical power of the study. In this instance, an opportunity presents whereby RCTs proceed with collection of its primary spirometry endpoints but is further supplemented or enhanced by RWD capturing hospitalisations and all-cause mortality. These methodologies, known as pragmatic or mosaic study designs, have potential to provide a broader spectrum of insights from across the different data sources while simultaneously generating evidence to appease the requirements of different decision-makers including regulators, HTA/payers and healthcare providers.

An alternative scenario of RCT/RWE endpoint misalignment relates to the practicalities of data collection and the instances where RCTs collect primary endpoints for which there is no readily available secondary data. RWD that is hard to access can be retrieved by primary data collection, but this does not account for occasions where equivalent RWD correlates fails to exist for an RCT endpoint altogether. This is helpfully illustrated by the field of patient-reported outcomes (PROs); a data collection methodology that is commonly appended to many RCT designs but not routinely available in real-world practice. Patient reporting, including symptom monitoring, has great potential to inform the care of patients while in receipt of a new investigational drug and there is an increasing body of evidence demonstrating its material clinical benefit [21]. The issue surrounding PROs is that they continue not to be routine practice for the most part, perhaps due to a burden of their collection, and are generally only implemented in clinical practice under special circumstances such as research or specific quality improvement initiatives.

At the front of attempts to organize PRO capture in the real-world is the use of technology-enable patient self-reporting. Electronic self-reporting platforms are being explored for prescription adherence [22] and adverse event monitoring [23] and provide an opportunity for collection of patient-centric metrics in the real-world. Herein, lies the future possibility of enhancing PROs driven RCTs with PROs captured in the real-world via electronic patient self-reporting, perhaps serving to enhance RCT generalisability and even reduce the data collection burden if implemented during a trial. Clearly, this proposition necessitates early evidence planning and upfront infrastructure investment from life science organisations, but it would represent a step towards the goal of generating broad and deep patient-centric evidence for consideration by all stakeholders.

Interestingly, through exploring synergy of RCT and RWE endpoints in the RCT environment, the discussion naturally takes a turn toward the issue of patient centricity. This is not surprising, given that patient-centric outcomes currently represent a sizable unmet need in the industry, leading to stakeholder calls for endpoints that are more at the core of what matters to patients. In this respect, and in keeping with the theme of this commentary, patient centricity signifies a domain where both RCT and RWE endpoints can shift together towards the goal of bettering outcomes for the patient. Table 1 lists several patient-centred endpoints by therapeutic area that are rarely used as common study endpoints but could have potential for captured by electronic self-reporting systems [24]. Again, such a commitment requires early planning in the research and clinical stages of evidence generation.

**Table 1.** Selected Patient-Centred Endpoints Across Several Different Therapeutic Areas (Non-exhaustive)

Therapy Area	Patient Centric Outcome Measures
Respiratory diseases	Dyspnoea, worsening disease, HRQoL, symptom control
Rheumatoid arthritis	Pain, Fatigue, Activity limitation, emotional and physical health impact, impact on work/home life
Diabetes	Psychological well-being, diabetes distress, depression
Cardiovascular disease	*Atrial fibrillation*: Ability to work, exercise tolerance, symptom severity, HRQoL *Heart failure*: Symptom control, activities of daily living, independence, psychosocial health
Lung cancer	HRQoL, fatigue and vitality, Pain, cough, shortness of breath, performance status
Breast cancer	HRQoL, arthralgia, neuropathy, vasomotor symptoms, fatigue, pain, depression, arm and breast symptoms, body image
Chronic kidney disease	Fatigue, pain, physical function, HRQoL
Inflammatory bowel diseases	Change in bowel symptoms, pain and discomfort, normal activities, energy and fatigue, weight
Major depressive disorder	Physical functioning, work functioning, social functioning, symptoms of depression, symptoms of anxiety

Source of information: The International Consortium for Health Outcomes Measurement

## Final Remarks

**Figure 2.**
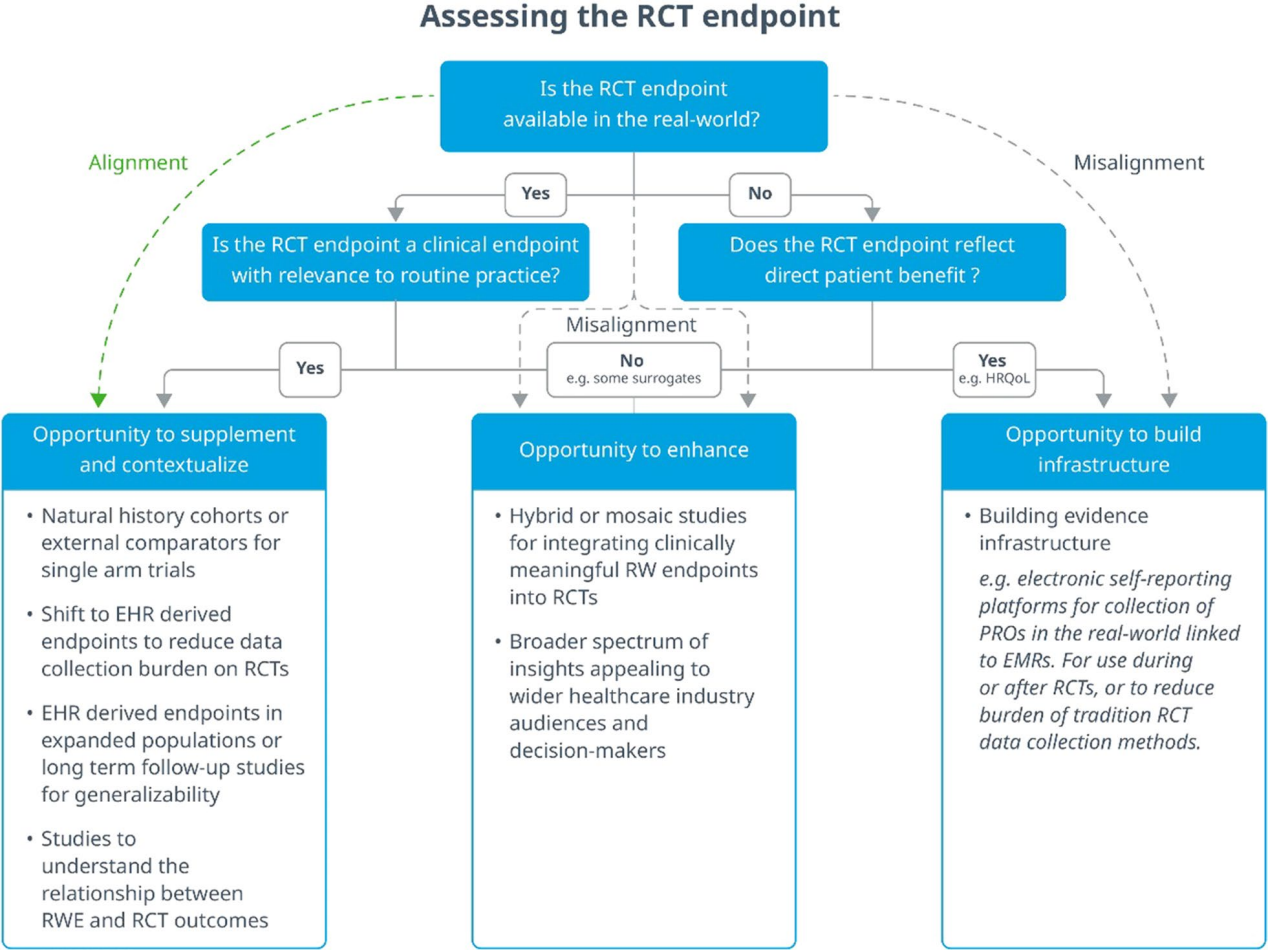
A Framework Proposing Several Pathways to Integrating RWE in the RCT Setting Based on the Clinical Relevance and Real-World Availability of Trial Endpoints. Trial endpoints that are relevant to routine practices, show a clinical benefit, and available in the real world represent an alignment with real-world study endpoints, and these RCTs could benefit from RWE supplementation or contextualisation. However, clinical trial endpoints that are not available in the real world or that do not measure direct clinical outcomes, or both, represent a misalignment with RWE and can benefit from hybrid methodologies or building real-world infrastructure

This commentary has described several opportunities for RWE to bridge several gaps in evidence generation present during drug clinical development and market access, with the goal of better meeting the endpoint expectations of industry decision-makers and stakeholders. In terms of clinical relevance, clinical benefit and RW availability, RCT and RWE endpoints will align in some circumstances but not in others, but across both instances there is opportunity to benefit from RWE in the RCT setting (Fig. 2). Here, the more clinical trials embrace RWD and RW endpoints, the more clinical development shifts towards accommodating for the concept of ‘totality of evidence’. In turn, this represents a desirable move towards being able to generate aspirational broad-spectrum evidence from a single RCT or study, that can satisfy multiple industry stakeholders in one sweep, from regulators to patients. Lastly, it was observed that both RCT and RWE endpoints, whether aligned or not, could do more to strengthen their patient centric focus. There is much value to be gained by putting the patients’ needs at the centre of evidence generation and this represents another way for the gap to be bridged between RCT and RWE endpoints.

Information Box **Provenance of data: real-world generated endpoints**

In RWD/E, many event-based endpoints are readily extractable or derivable from EMR and registry data due to the implementation of structured electronic coding systems in healthcare centres, providing that care for the events of interest are provided by the institutions generating these data [25]. These coding systems are particularly useful to disease event endpoints because they allow outcomes to be derived from across diverse secondary data sets such as claims information. In terms of mortality endpoints, RWD sources may be linked to national death records or other sources when they are not present in the EMRs, which is often the case when death occurs outside the treating hospital or institution.

Yet it is often observed that secondary data alone does not provide RCT endpoint correlates equally as effectively across all therapeutic areas; a reminder that clinical investigation is not the principal intention of secondary data collection. The diagnostic coding ontologies of secondary data sources can be misaligned between centres or systems and can sometimes themselves fail to capture the complex nature of diagnoses [26], with algorithms designed to identify disease events being evaded by disease pathway complexities. On top of this, consistency of reporting is essential, as the accuracy of secondary real-world data is truly in the hands of the people using these coding systems with practices likely to vary.

Under many of these restrictions, primary real-world data collection through traditional chart review approaches can support the derivation of complex endpoints in the real world that reside in non-structured data such as HCP notes, although some benefits associated with RWD collection, namely speed and cost effectiveness, are sacrificed. Here however, natural language processing and other machine learning methodologies are increasingly capable of superseding primary data extraction and can automate the collection of hard-to-access clinical information relating to key patient-provider interactions in routine clinical practice.
